# Toxic Relationships Described by People With Breast Cancer on Reddit: Topic Modeling Study

**DOI:** 10.2196/48860

**Published:** 2024-02-23

**Authors:** Cara Anne Davidson, Richard Booth, Kimberley Teresa Jackson, Tara Mantler

**Affiliations:** 1 Department of Health and Rehabilitation Sciences Faculty of Health Sciences Western University London, ON Canada; 2 Arthur Labatt Family School of Nursing Faculty of Health Sciences Western University London, ON Canada; 3 School of Health Studies Faculty of Health Sciences Western University London, ON Canada

**Keywords:** breast cancer, intimate partner violence, meaning extraction method, Reddit, sentiment analysis, social media, social support, toxic relationships, topic modelling

## Abstract

**Background:**

Social support is essential to promoting optimal health outcomes for women with breast cancer. However, an estimated 12% of women with breast cancer simultaneously experience intimate partner violence (IPV; physical, psychological, or sexual abuse by an intimate partner). Women who experience IPV during breast cancer may lack traditional social support, and thus seek out alternative sources of support. Online community forums, such as Reddit, can provide accessible social connections within breast cancer–specific communities. However, it is largely unknown how women with breast cancer use Reddit to describe and seek support for experiences of IPV.

**Objective:**

This study aims to explore how patients with breast cancer describe toxic relationships with their partners and immediate family members on Reddit.

**Methods:**

This exploratory, cross-sectional, topic-modeling study analyzed textual data from 96 users in the r/breastcancer subreddit in February 2023. The meaning extraction method, inclusive of principal component analysis, was used to identify underlying components. Components were subjected to sentiment analysis and summative content analysis with emergent categorical development to articulate themes.

**Results:**

Seven themes emerged related to toxic relationships: (1) contextualizing storytelling with lymph nodes, (2) toxic behavior and venting emotions, (3) abandonment and abuse following diagnosis, (4) toxic relationships and social-related fears, (5) inner strength and navigating breast cancer over time, (6) assessing social relationships and interactions, and (7) community advice and support. Toxic relationships were commonly characterized by isolation, abandonment, and emotional abuse, which had profound emotional consequences for patients. Reddit facilitated anonymous venting about toxic relationships that helped patients cope with intense feelings and stress. Exchanging advice and support about navigating toxic relationships during breast cancer were core functions of the r/breastcancer community.

**Conclusions:**

Findings emphasized the value of Reddit as a source of social support for patients with breast cancer experiencing toxic relationships. Clinicians who understand that many patients with breast cancer experience toxic relationships and considerable psychological sequelae are better prepared to support their patients’ holistic well-being. Further investigation of Reddit as a possible resource for advice, information, and support has the potential to help inform clinical practice and subsequently, patient health outcomes.

## Introduction

Breast cancer has a way of making existing cracks in relationships even wider. Just like water will fill a crack in the road, freeze, and create a larger gap, breast cancer tends to permeate all parts of our lives and distance us from people with whom we have troubled relationships.

Original poster #85

Projected rates of breast cancer in Canada have remained consistent over the past 5 years, with estimates that approximately 1 in 8 women will develop breast cancer in their lifetime and breast cancer will account for 25% of all new cancer cases [1-3]. Women’s experiences of breast cancer are influenced by the social determinants of health, particularly their social environment [4,5]. Among patients with breast cancer, strong social relationships have been found to act as a buffer to stress [6] and help to improve treatment effectiveness, psychological functioning, coping, survival, and quality of life, as well as prevent cancer recurrence [7-9]. Conversely, weak or nonexistent social relationships have been broadly linked to long-term psychological distress [10] and an increased risk of breast cancer progression, recurrence, and mortality [11,12]. However, there is a need for research that explores connections between social relationships and breast cancer outcomes among diverse populations and social contexts.

Intimate partners (eg, spouses and significant others) and immediate family members (eg, parents and siblings) are perceived as the most important social supports for patients with breast cancer [13,14], as they provide essential social-emotional, tangible, affection, and positive social interaction support [15]. For example, partners commonly serve as the primary caregivers of patients with cancer [16]. However, not all social relationships are supportive [17]. Patients who experience intimate partner violence (IPV) may face a lack of support due to the abusive behaviors of their partner [18]. IPV, understood as physical, psychological, or sexual abuse within the context of coercive control by an intimate partner [19], concurrently affects an estimated 12.5% of patients with breast cancer [20]—and this is likely to be an underestimation given underreporting of IPV [21]. Similarly, patients may be negatively affected by an unsupportive (but not necessarily abusive) partner [22], as well as abusive or unsupportive family members [23,24]. Aside from the patients themselves, immediate female family members are often most affected by a breast cancer diagnosis; unsupportive reactions often include being in denial about the diagnosis and abandoning the patient [25].

Toxic relationships are characterized by conflict, competition, undermining, disrespect, and a lack of cohesiveness [26]. Toxic relationships encompass unsupportive and abusive dynamics in both romantic (eg, a partner) and platonic (eg, a family member) contexts and are associated with emotional distress [26], which imparts numerous downstream mental and physical health consequences [27]. To compensate for unmet support needs, patients with breast cancer may expand their social networks via the internet, including social media [28]. Online forums are a popular means of accessing information and support related to breast cancer awareness, literacy, and treatment [29-33]. The use of online breast cancer forums grew exponentially between 2006 and 2010, growing from an estimated 282,000 new posts per year to over 1,270,000 new posts per year [34] and continues to increase over a decade later [35,36]. Despite data availability and the potential for knowledge advancement [33], research on patient social media use, particularly in the context of toxic relationships, is underexplored.

Reddit, the world’s third most popular social media platform, is an online forum dedicated to community-building, news dissemination, and discussion facilitation [37]. The Reddit platform consists of topic-specific subreddits (ie, forums), where all content is user-generated. Users subscribe to subreddits that interest them to see more related content. Users can post content, as well as comment and vote on others’ content. To join Reddit, users create a username and password—no identifiable information is required. Reddit’s capacity for anonymous participation and long-form, conversational content makes the platform a rich source of self-reported textual data [38]. The Reddit platform includes breast cancer–specific spaces that offer access to psychosocial support (eg, r/breastcancer), presenting a unique and valuable opportunity to explore how patients with breast cancer navigate toxic relationships after diagnosis. Previous research has provided preliminary insights into how patients with breast cancer use Reddit [39], but there is a notable gap in the literature regarding how patients with breast cancer describe toxic relationships and their psychosocial impacts on Reddit. Studying social media data has the potential to generate significant advances in knowledge [33], which can inform improvements to psychosocial support for patients with breast cancer experiencing toxic relationships and enhance care providers’ ability to promote patient well-being. Accordingly, this study sought to explore how people with breast cancer describe toxic relationships with their partners and immediate family members on Reddit.

## Methods

### Design

This exploratory, cross-sectional, topic-modeling study was conducted from December 2022 to February 2023 and aimed to explore how patients with breast cancer describe toxic relationships with their partners and immediate family members on Reddit. As of February 2023, the public r/breastcancer subreddit, established in 2011, included 13,900 subscribers and self-identified as a support and information group for people who have been diagnosed with breast cancer and their caregivers and loved ones. While Reddit generally attracts young White men of high socioeconomic status [38], demographics vary by subreddit and r/breastcancer is hypothesized to be largely composed of women [40].

### Ethical Considerations

This study was deemed exempt from oversight by the author’s institutional ethics review board because all data were gathered from the public domain (per Article 2.2 of the Tri-Council Policy Statement on Ethical Conduct for Research Involving Humans). The subreddit at the center of this study was public at the time of data collection and writing, meaning that any person could access its content at any time. It was therefore determined that r/breastcancer users had no expectation of privacy, negating the need for oversight by an ethics review board.

### Data Collection

This subreddit was scraped for textual data from posts and comments using the Python Reddit application programming interface wrapper. No date limits were imposed. An iterative approach to keyword-based searching extracted posts (n=187) related to toxic relationships with partners and immediate family members. Two keyword strings were combined to scrape data: String 1 included words associated with a toxic relationship (eg, narcissist, boundaries, abuse, violence, assault, unsupportive, cheater, affair, divorce, toxic, abandon, and manipulate) and string 2 included words that identified people of interest in the immediate family of the user (eg, abuser, spouse, partner, marriage, significant other, parent, and sibling). To be scraped, posts were required to include a minimum of 1 keyword from both string 1 and string 2 (see Textbox 1).

**Keywords included in the final iteration of the search strategy.**

**String 1**

narcissist; boundaries; abuse; abusive; abusing; abused; violent; violence; assault; assaulting; assaulted; harass; harassed; harassing; lie; lied; neglect; unsupportive; not supportive; not supporting; no support; cheater; cheated; cheating; affair; divorce; divorcing; break up; breaking up; broke up; toxic; abandoned; manipulate; manipulated; emotionally unavailable; disown; alone; selfish; strained

**String 2**

abuser; husband; wife; partner; hubby; marriage; girlfriend; boyfriend; gf; bf; SO; significant other; spouse; mom; mother; mum; dad; father; parent; parents; sibling; sis; sister; brother

**Figure 1:**
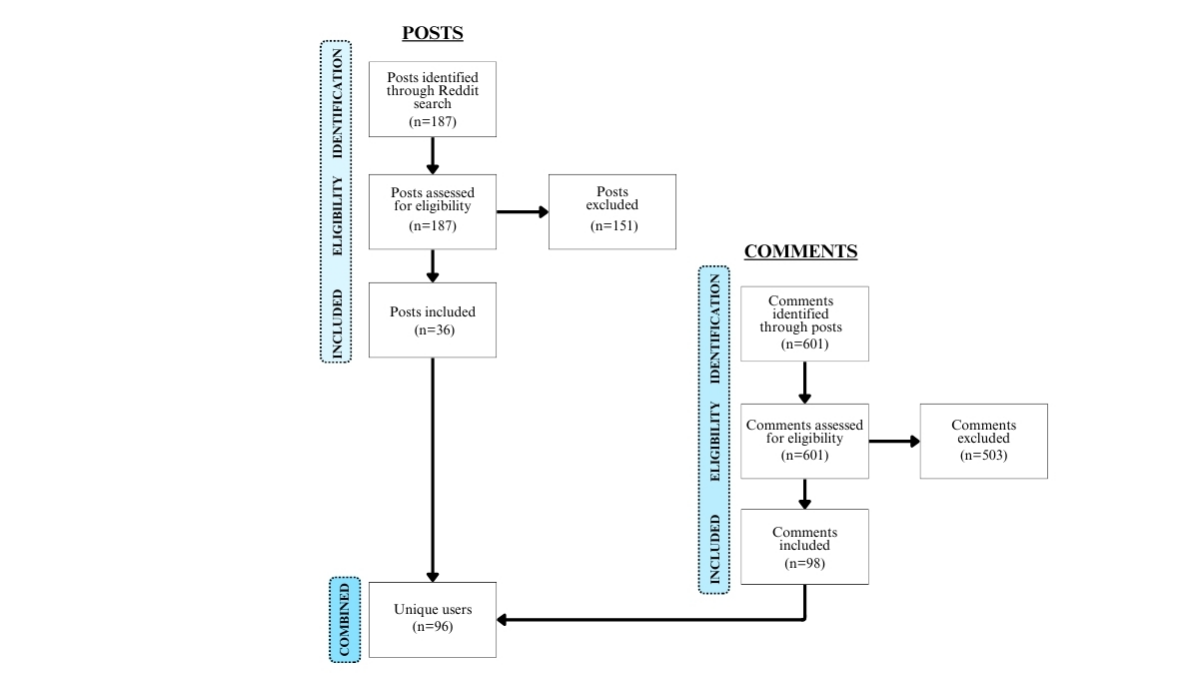
Final data corpus diagram.

The scraped posts were then screened for eligibility by one of the authors (CAD), such that posts were ineligible if they addressed anyone other than a partner or immediate family member, were of an administrative nature posted by a moderator, were posted by a user who did not have breast cancer, aimed to exclusively seek or share medical information, or described toxic relationships outside of the context of breast cancer. After screening, 36 posts were eligible for inclusion. Eligible posts were scraped for comments (n=601), of which 98 were relevant (as determined by CAD using the eligibility criteria described above used to filter posts). Textual data were compiled into packets, where 1 packet represented the total relevant contributions (ie, posts and comments) from a single user, with an average of 260 words per packet. The final data corpus included 96 unique users with 36 posts and 98 comments (see Figure 1).

Reddit users have no expectation of privacy in public subreddits and have agreed to the platform’s end user license agreement; all Reddit user content is subject to use by third parties at any time [41]. However, recommended ethical practices aim to protect participant privacy by censoring usernames and avoiding direct quotes through exclusion or paraphrasing to prevent reverse-searching [38,42]. Accordingly, within this study, users were assigned an original poster (OP) number, and reported quotes were reworded to convey their original meaning and style but protect the OP’s identity. For example (fictitious), “My mom has *never* bothered checking on me” could become “My mother doesn’t *ever* ask how I’m doing.”

### Analysis

#### Multistaged Approach

**Figure 2:**
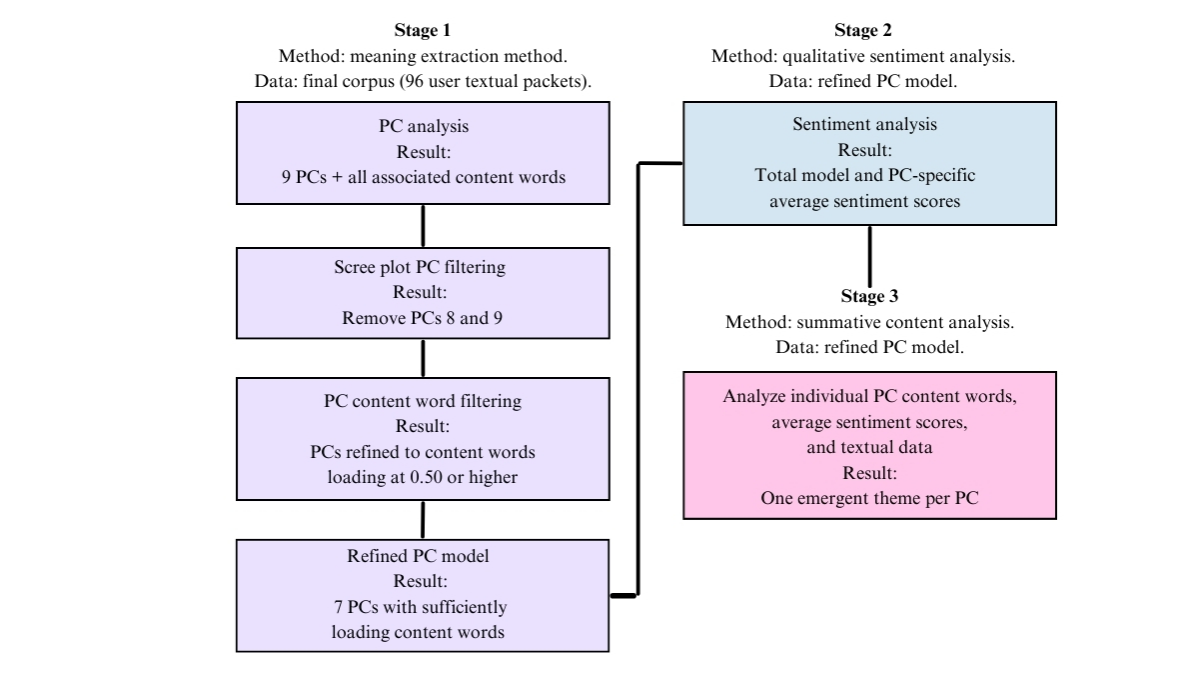
The 3 stages of analysis. PC: principal component.

A 2021 systematic analysis by Proferes et al [39] identified that computational-driven textual analysis (which includes topic modeling) was the primary means of knowledge generation using Reddit data. However, the authors also identified that such analyses are enhanced by the addition of qualitative and mixed methods analyses that account for contextual details [39]. Accordingly, the data corpus was subjected to a 3-stage, mixed methods analysis that used (1) the meaning extraction method (MEM), (2) qualitative sentiment analysis, and (3) summative content analysis (Figure 2).

#### Stage 1: Meaning Extraction Method

The MEM is a form of topic modeling useful for social media data exploration [43] and for generating large sample sizes of participants that are traditionally difficult to recruit [38]. Within other breast cancer–related research using Reddit data, the MEM has been described as a cost-effective means of identifying common themes described by patients [39]. The results of this method have been found to be similar in content and utility to those of traditional research methods in this domain (focus groups) [39].

The MEM identifies word clusters that co-occur in a data corpus, providing an efficient means of extracting meaningful patterns in language within high volumes of natural language data [38,43]. The Meaning Extraction Helper developed by Boyd [44] was used to analyze the textual packet data corpus, inclusive of the removal of common closed and open class words (<7.5%) and content word retention (≥5%), producing a binary output of each retained content word per OP (eg, 0=absent and 1=present). Boyd [45] also developed an open-access script for the R open-access statistical software (R Foundation for Statistical Computing), which was adapted for a principal component analysis (using a varimax rotation [43]). This produced a 9-component model that was considered acceptable (*K*^2^=3357.40, *df*=304, *P*<2.2e–16, and KMO=0.538 [43]). Using a scree plot analysis, components 1 to 7 were retained. The 7 retained components explained 84.16% of the variance—a high proportion for a natural language application [43]. A high loading threshold of 0.50 was imposed on the content words within each component to promote thematic clarity and reduce cross-loading. The final components with refined content words were considered sufficiently strong (≥3 content words per component [46]).

#### Stage 2: Qualitative Sentiment Analysis

Qualitative sentiment analysis aims to assess the affective valence of components and their content words [47]. Modern qualitative sentiment analysis (ie, internet-based) is an increasingly popular and effective method of interpreting user-generated social media content [48]. Using the *syuzhet* R package [49] and Afinn sentiment lexicon of –5 (negative sentiment) to 5 (positive sentiment [50]), a total model and 7 component-specific sentiment scores were computed based on content words. The content word *tchp* was changed to *combination drug cancer therapy* for the algorithm because it cannot assess acronyms.

#### Stage 3: Summative Content Analysis

Summative content analysis with emergent categorical development was used to articulate patterns and themes within the textual data packets for each of the 7 refined components [51]. Component categories (referred to as themes) were inductively developed to describe their overall message, inclusive of the use of sentiment scores to contextualize positioning. The 6-step approach to trustworthy thematic analysis by Nowell et al [52], rooted in the trustworthiness theory of Lincoln and Guba [53,54] was adopted.

#### Summary of Analysis

Quantitative topic modeling (with MEM) was combined with qualitative sentiment and content analysis to produce a comprehensive analytical framework capable of providing an overall interpretive assessment of the data corpus. The r/breastcancer subreddit includes thousands of textual data sources, requiring the combination of complex methods to efficiently target and isolate meaningful, manageable patterns from the large volume of natural language data [38,43]. The MEM is a computational method specifically developed to facilitate efficient filtering of large textual data sets, however, a second stage of qualitative or mixed methods–based analysis is recommended to facilitate deeper exploration and interpretation in context [39]. Accordingly, sentiment analysis was applied within MEM-generated principal components to facilitate the assessment and incorporation of considerations of user’s emotions and situational contexts. Following MEM and sentiment analysis, content analysis was used to deeply explore principal components through the lens of their socioemotional contexts to enrich interpretation and understanding. In sum, this combined mixed methods framework aimed to produce holistic, contextualized insights from MEM-generated categories, which is well-suited to complex, dynamic social media data.

## Results

### Overview of Themes

Seven distinct but related themes emerged from descriptions of toxic relationships by patients with breast cancer on Reddit, presented in order of explained variance proportion (highest to lowest) as follows: (1) contextualizing storytelling with lymph nodes, (2) toxic behavior and venting emotions, (3) abandonment and abuse following diagnosis, (4) toxic relationships and social-related fears, (5) inner strength and navigating breast cancer over time, (6) assessing social relationships and interactions, and (7) community advice and support. The overall corpus sentiment score was –4, indicative of very negative sentiment. Theme-specific sentiment scores (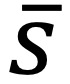) reflect the average valence of retained content words within each component.

### Theme 1: Contextualizing Storytelling With Lymph Nodes

I’ll have to get my lymph nodes removed next, among other things. Treatment is lonely and miserable.

OP 2

The first theme was classified as neutral (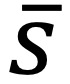
*=*0.00) and included *lymph*, *node*, and *pick* as key content words. Lymph nodes functioned as context indicators in users’ stories about toxic relationships to highlight their temporality within cancer treatment. For example, one user was undergoing chemotherapy while navigating a toxic relationship with their mother. This OP prefaced their post by sharing, “After a lot of treatment, my cancer went from grade 3 to grade 1. My lymph nodes shrunk as well” (OP 66).

They then went on to disclose unsupportive behavior from their mother, stating, “My mom doesn’t think I’m capable of making my own decisions–but I am. I’ve picked excellent physicians and made it to all of my appointments” (OP 66).

### Theme 2: Toxic Behavior and Venting Emotions

I’m going to vent because I think it’s better to write than to cry...

OP 65

The second theme was classified as neutral (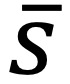
=0.00) and described toxic relationships that the user experienced a strong emotional reaction to, which prompted them to vent their emotions on Reddit. Key content words included *boundary*, *effort*, *vent*, *upset*, and *stress*. Users reported a variety of toxic behaviors, such as boundary violations and disrespectful or abusive actions. Venting was commonly used to cope with powerful negative emotions associated with toxic relationships.

Users felt unsupported when their partners or families reacted to their diagnosis by becoming detached or distressed to the extent of relying on the patient for support. To illustrate, one user expressed disappointment in their father’s silence after diagnosis, stating, “My dad isn’t there for me. I guess I shouldn’t be surprised, he’s always been like this” (OP 45).

Other users were frustrated with bearing the emotional burden for others regarding their cancer. For example, one OP resented their husband for expecting them to manage his emotions, sharing, “I did my best to explain that I needed him to be my rock. He got upset... he wanted us to be mutually supportive. But he doesn’t have cancer.... I do!” (OP 62).

Some OPs described being disrespected and emotionally abused following their diagnosis. For instance, one OP shared that their partner told them, “Lately, you aren’t sexually desirable to me without your natural breasts. I miss them and how they felt... probably even more than you do” (OP 65).

Similarly, another OP disclosed experiencing emotional and verbal abuse from their partner both before and after their breast cancer diagnosis. This OP shared feeling extremely upset that just 2 weeks after their diagnosis, their partner asked them, “How long are you going to pull the breast cancer card?” (OP 85).

Toxic relationships described within this theme were strongly associated with venting, that is, posting negative, emotionally charged content. For example, an OP trying to cope with being isolated by their family prefaced their story by writing, “Heads up that this is a massive, sad vent post. Sorry but I feel like I need to shout into the void” (OP 34).

### Theme 3: Abandonment and Abuse Following Diagnosis

Anyone else dealing with an emotionally abusive spouse before and during cancer? I’m trying to get away and he’s being awful.

OP 40

Theme 3 was classified as slightly negative (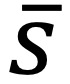
=–1.00) and captured how patients in toxic relationships were abandoned or emotionally abused by their partners following their diagnosis. Key content words included *devastate*, *experience*, and *abuse*. Patients who navigated abandonment or abuse concurrently with a breast cancer diagnosis reported feeling emotionally devastated.

Abandonment was especially common after disclosing a breast cancer diagnosis. For example, one OP shared that their husband abandoned them on the way home from their diagnosis appointment, stating, “He said he won’t look after the kids and plans on leaving” (OP 9). Other users were abandoned as treatment began. Many users who shared stories of abandonment described emotional whiplash, characterized by a sudden, unexpected transition from feeling secure in their relationship to feeling betrayed following abandonment. As illustrated by one user, “He made me feel cared for, loved, and safe... until I said I was considering a mastectomy. Then he shut me out” (OP 12).

The emotional impacts of betrayal were devastating. An OP whose long-term partner unexpectedly broke their promise to stick by them during treatment shared, “I am completely devastated. I am infuriated. He and my body betrayed me. I am so furious” (OP 86).

Of partners who stayed following a diagnosis, many subjected the patient to emotional abuse. One OP was told that they deserved their cancer, recounting, “He used my cancer against me by saying I got it because I’m weak and that’s just natural selection at work. He told me not to bother with treatment and to just let nature run its course” (OP 76).

Other experiences involved infidelity, threats of child abandonment, accusations of faking symptoms, and coercion in treatment choices. Emotional abuse was repeatedly described as devastating. For example, an OP whose spouse had been emotionally abusive for years posted, “What can I do to stop feeling devastated that my husband feels I should be punished all the time?” (OP 40).

### Theme 4: Toxic Relationships and Social-Related Fears

Do any of you also feel like the emotional consequences of breast cancer are almost worse to deal with than the physical?

OP 66

The fourth theme was classified as slightly negative (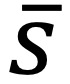
=–0.75) and focused on social-related fears associated with breast cancer. Key content words included *biopsy*, *tchp*, and *scare*. Patients’ fear stemmed from anticipating or experiencing a negative reaction to their breast cancer by a toxic family member or partner. For example, an OP who disclosed a toxic family shared dreading their reaction to their cancer, expressing, “The fear of how my family will react to my breast cancer diagnosis is nearly as overwhelming as the actual diagnosis” (OP 81).

Other users felt scared because they had already experienced an unsupportive reaction by a toxic family member or partner to their cancer. For example, one OP felt scared and hopeless after being gaslit by their partner about their diagnosis, sharing, “He was trying to tell me that my breast cancer was all in my head, despite having seen my biopsy results and meeting with multiple members of my medical team” (OP 3). Similarly, an OP whose family neglected to support them after learning of their diagnosis expressed, “My family doesn’t care about me or my breast cancer. It makes me feel scared and alone” (OP 34).

### Theme 5: Inner Strength and Navigating Breast Cancer Over Time

I thought to myself that if my cancer ever came back, I’d rather deal with it alone than with a person like that.

OP 32

Theme 5 was classified as slightly positive (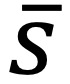
=1.00) and highlighted how breast cancer was disruptive to the lives of patients. Key content words included *future*, *matter*, and *strength*. Users described how health and social adversity influenced their inner strength. Toxic relationships that emerged after diagnosis were especially trying for patients. For instance, one OP expected their partner’s support as they began cancer treatment (as their partner had promised). However, the OP’s partner abruptly took back their commitment, leaving the OP to navigate cancer alone: “They sent me a message the next day and said they don’t want anything to do with me” (OP 12).

Inner strength emerged as a dynamic construct that was both challenged by experiencing a breast cancer diagnosis and toxic relationships and enhanced by surviving these adverse experiences. Many users believed that surviving breast cancer concurrently with exposure to toxic relationships was a testament to their inner strength. For example, one OP attributed their inner strength to recovering from breast cancer while navigating a lack of empathy and support from their spouse. This OP stated, “I feel 100% confident that I am a strong, intelligent woman who can face almost anything” (OP 64), while sharing that they had received a new cancer diagnosis. Inner strength also enabled users to regain a sense of control over how they were going to navigate living with a breast cancer diagnosis. For example, an OP who was abandoned by their partner after being diagnosed stated, “I finally felt strong enough to delete his contact information because I couldn’t stop myself from calling him–it was the best choice I could have made” (OP 47).

### Theme 6: Assessing Social Relationships and Interactions

I am immensely grateful for you all for helping me navigate a chaotic and frustrating moment.

OP 85

The sixth theme was classified as marginally positive (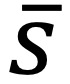
=0.20) and described how OPs assessed their social relationships and interactions. Key content words included *conversation*, *response*, *listen*, *regret*, and *grateful*. Users assessed the quality of social support from family based on whether they felt judged, subjected to toxic positivity, or made to listen to unsolicited advice. For example, an OP with an emotionally unsupportive family shared, “I think a lot of family think it’s helpful when they shove positivity down our throats. What we really need is support and someone to listen without trying to solve all our problems” (OP 82).

For some users, responses to breast cancer unveiled toxic relationships that they regretted having to face. For example, an OP with unsupportive parents shared, “I regret that my breast cancer forced me to confront that my parents never have and still don’t support me how I need them to” (OP 4). However, OPs who discovered both toxic and supportive relationships during cancer expressed gratitude for the sources of support they did have. As one OP stated, “Sometimes I get jealous of people whose parents love and support them, but then I remember the rest of my friends and family who showed up for me when I needed them, and I’m grateful” (OP 32). The subreddit community was repeatedly praised by users because it was such a valuable source of support. For instance, one OP shared, “I am endlessly grateful for the knowledge and resilience of this community” (OP 53).

### Theme 7: Community Advice and Support

I know what it feels like to be abandoned. I could tell you all the red flags in a man’s behavior... but just trust me–it’s better to be alone. You dodged a MASSIVE bullet. A person who lacks compassion about your breast cancer is NOT a good life partner. Please message me if you need someone to vent to. I really do understand...and you’ve got this.

OP 89

The seventh theme was classified as marginally negative (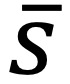
=–0.17) and characterized a core function of r/breastcancer: providing advice and support. Key content words included *money*, *quit*, and *follow*. The subreddit facilitated advice regarding various topics, especially related to navigating financial matters and treatment options in the context of a toxic relationship.

Numerous users offered money-related advice to OPs facing difficult financial situations because of toxic relationships. Situations included financial coercion, exploitation, and manipulation following cancer disclosure and managing finances during separation from a toxic partner. For instance, one OP was abandoned by their partner during a joint real estate purchase. A community member with self-professed real estate expertise strongly advised the OP against continuing with the investment, writing, “I’m begging you... please do NOT sign anything else! Lose your money... that’s not important... please do not continue with this purchase” (OP 89).

Members also counseled OPs about postmastectomy reconstruction by offering advice on how to reduce social pressure and prioritize personal preferences. For example, one OP shared how they resisted their partner’s pressure to follow reconstruction, stating, “I made him look at photos of reconstruction to show him that it’s not a free boob job and can be ugly. He changed his tune real quick” (OP 39).

Members who were ultimately pressured into reconstruction strongly encouraged OPs to follow their instincts. For example, one member who was coerced into reconstruction by their husband advised, “I constantly wish I went flat instead. If I had to do it again I would listen to my gut and go flat” (OP 8).

Similarly, it was common to share advice about treatment adherence. Many OPs struggling with a lack of support expressed wanting to quit treatment. While members empathized with users and understood their feelings, they ultimately encouraged continuing. For example, one OP shared, “I’m just sick of this. I’m pretty sure I’m done with it all” (OP 34).

The community offered empathy, such as, “When I was in the middle of your treatment, I was frustrated too and tried to quit every week” (OP 7), as well as advice, for example, “Don’t stop treatment without a good reason. It’s a gift in spite of tough side effects because it keeps us alive” (OP 48).

## Discussion

### Principal Results

This study explored the use of the r/breastcancer subreddit by patients to describe toxic relationships with their partners and immediate family members. Themes highlighted patients’ lived experiences of toxic relationships, emotional impacts, and support from the subreddit community. A key finding was that many people with breast cancer sought out the r/breastcancer subreddit to share their experiences of toxic relationships, often including descriptions of abandonment, isolation, and emotional abuse within this context. Further, this study presented compelling evidence that toxic relationships impart profound emotional consequences for patients and that some patients cope with these strong emotions through online venting. This work also emphasized the value of online communities like Reddit as alternative, complementary sources of support for patients experiencing toxic relationships.

### Comparison With Prior Work

#### Abandonment and Betrayal as Common Experiences

These findings suggest that abandonment is a common experience for patients with breast cancer following diagnosis. Prior research has lacked consensus regarding the risk of abandonment among patients with breast cancer after diagnosis [55,56]. Generally, however, women are more likely to be abandoned by a partner after being diagnosed with a serious medical illness [57]. Further, distancing is the most prevalent unsupportive response experienced by a patient following their breast cancer diagnosis [23]. Fears and feelings of abandonment following diagnosis are also well-documented within breast cancer research [58-60]. Given this understanding, and considering that Reddit data can be regarded as an authentic representation of user experiences [61], it is reasonable to conclude that these findings are suggestive of an increased risk of abandonment for patients with breast cancer.

A novel finding was the occurrence of emotional whiplash, where a patient was initially promised support by their partner but was later abandoned unexpectedly. The emotional transition from security to betrayal was repeatedly reported as devastating. There is limited research describing betrayal in the context of abandonment and breast cancer, but it is known that feelings of betrayal in this context can reduce the desire for future relationships [62]. Broadly, the loss, disruption, and deterioration of social ties are some of the most stressful experiences a patient with cancer can face [6], which makes abandonment a serious risk factor for reduced mental health [63]. Comprehensive cancer care entails stress-reducing psychosocial interventions [63], but a limited understanding of the psychological effects of betrayal hinders clinicians’ ability to optimally manage abandonment-related stress.

#### Anonymous Venting Enables Disclosure of Toxic Relationships

The central role of venting within the r/breastcancer community highlighted the unique socioemotional needs of patients with breast cancer in the context of toxic relationships. Toxic relationships impart emotional consequences that can be difficult to navigate and cope with [26]. Venting is a disinhibitory, emotion-focused strategy for coping with stress [64,65]. Venting can be considered a form of expressive writing, that is, writing that describes a deeply personal experience [66], which is well-evidenced to facilitate coping with psychological distress [67]. Online venting was consistently described as cathartic among patients in this study, aligning with prior evidence of patients with breast cancer seeking support in online communities during periods of stress [68] and perceiving reduced stress after they vent online [69]. Further, patients with breast cancer who self-manage their emotions by narrating their experiences are known to experience strong psychological benefits [70].

It might be expected that the stigma attached to breast cancer and toxic relationships would hinder disclosure [58,71], however, seeking out group-oriented support is reportedly most common for diseases considered stigmatizing [72]. The latter position is consistent with this study, as venting posts often included stigmatized thoughts and feelings (eg, wanting to ‘give in’ to cancer or discussing abuse without wanting to leave the relationship). Further, it appeared that Reddit’s capacity for anonymity created a sense of safety that made patients comfortable disclosing information considered stigmatizing, which is consistent with existing evidence [73]. Overall, patients appeared to perceive anonymous venting via Reddit as an effective, safe strategy for coping with stress from toxic relationships. Interventions that aim to promote coping among this patient population would likely benefit from integrating anonymity to encourage uninhibited self-expression.

#### Advice About Navigating Toxic Relationships

Validating the feelings of other users, as well as soliciting and providing advice regarding toxic relationships, were core activities within r/breastcancer. It was previously known that participation in online forums contributes to the practical, informative, and emotional empowerment of patients with breast cancer [74]. However, this study uniquely identified that community members on Reddit often urged OPs to leave or go against the wishes of their abusive partner. While well-intentioned, this advice may not always be safe or practical. Leaving an abusive partner can be the most dangerous time in the relationship due to an increased risk of retaliation [75]. Similarly, acting in a manner that might antagonize an abuser can initiate or escalate relationship discord and consequently increase the risk of violence [76]. Furthermore, patients who depend on an abusive partner (eg, for caregiving, access to health insurance, and transportation to appointments [77]) may be unable to leave or risk the relationship by acting defiantly [78]. Resultantly, relationship advice received on Reddit by patients with abusive partners may have been incompatible with their reality or suboptimal in promoting their safety.

This indicates a knowledge gap concerning safety planning within r/breastcancer; safety planning can be understood as the development of strategies to reduce the risk of abuse and enhance support [79]. Safety planning is a proven, widely endorsed health promotion intervention that is effective both within an abusive relationship and after leaving [80,81]. Considering the prevalence of abuse among patients with breast cancer [20] and that many seek support in online forums such as Reddit [39], it could be useful to raise awareness of safety planning within r/breastcancer as a health promotion strategy. Further, considering the importance attributed to inner strength by patients in this study, building awareness of strengths-based approaches to safety planning [82] could be particularly useful. For example, community moderators could pin relationship-related resources (eg, hotlines and informative websites) as the top comment under posts about challenging, potentially toxic relationships. However, a needs assessment would be best suited to developing an IPV-related intervention considered acceptable and effective within r/breastcancer.

### Clinical Implications

Psycho-oncology care teams play a critical role in optimizing health outcomes for patients with breast cancer, yet the emotional well-being of patients with cancer is often underreported and underexplored [83]. Patient-reported social media data offers real-time insights into patient experiences and needs which can be beneficial for informing clinical practice [33,83].

Clinicians who understand that many of their patients with breast cancer are negatively affected by toxic relationships are better prepared to support their emotional well-being. Acquiring knowledge about practices and resources that foster coping and inner strength, including venting and safety planning, can contribute to improved patient outcomes.

Some clinicians may be unfamiliar with the advantages of online forums for patients, but recognizing the potential benefits could enhance care [84]. Recommending Reddit as a possible source of advice, information, and support could be a valuable addition to clinical practice for patients navigating breast cancer and toxic relationships. However, because digital literacy is often overlooked in breast cancer care [85], clinicians who concurrently promote digital literacy can empower their patients to access online communities and ultimately, improve their health outcomes.

### Limitations

There are limitations to this work. First, the analysis was conducted by a single researcher, which may have introduced bias in data interpretation. The analysis also relied heavily on automated methods that may have been inadequate in fully capturing nuance or interpreting context cues in textual data. Second, these data are self-reported, which may have resulted in biased perspectives. While users in this sample self-identified as patients with breast cancer, it was not possible to validate this. These data may have inadvertently included content from online robots or people without breast cancer, and thus may not accurately reflect the experiences of the target population. Additionally, these data were scraped from a single social media platform and may not be representative of the experiences of patients who use other social media platforms, do not use Reddit to discuss their personal lives, or lack access to an internet-enabled device. No demographic information was available to further contextualize findings. It is important to note that these results only relate to experiences of emotional abuse, as physical and sexual abuse were not represented in the data. Furthermore, all participants could write in English, were digitally literate, and had access to the internet, meaning that the findings may not represent the experiences of patients who are nonanglophone or lack technological access or literacy. Caution should be used when applying these findings to other patients with breast cancer.

### Conclusions

This study identified that toxic relationships described by patients with breast cancer on Reddit were common and characterized by abandonment, abuse, and unsupportive behaviors. Patients often experienced profound emotional reactions to this form of social stress and anonymous venting on Reddit was described as an effective coping mechanism. Some patients described breast cancer and toxic relationships as adverse experiences that ultimately enhanced their inner strength. Overall, the r/breastcancer community appeared to be a means of exchanging advice, information, and support for patients experiencing toxic relationships. Clinicians who understand that their patients may be negatively affected by toxic relationships are better prepared to support their holistic well-being. Further investigation of Reddit as a possible source of advice, information, and support has the potential to help inform clinical practice and subsequently, improve patient health outcomes.

## Abbreviations

IPV: intimate partner violence
MEM: meaning extraction method
OP: original poster

## Data Availability

The data sets generated or analyzed during this study are available from the corresponding author on reasonable request.
